# Loss of *LXN* promotes macrophage M2 polarization and PD-L2 expression contributing cancer immune-escape in mice

**DOI:** 10.1038/s41420-022-01227-7

**Published:** 2022-11-03

**Authors:** Yaping Li, Yanhui Tan, XiuZhen Li, Xuanming Chen, Lingzhu Wang, Lijun Zhang, Shaohua Xu, Kebing Huang, Wei Shu, Hong Liang, Ming Chen

**Affiliations:** 1 State Key Laboratory for Chemistry and Molecular Engineering of Medicinal Resources, Key Laboratory for Chemistry and Molecular Engineering of Medicinal Resources (Ministry of Education of China), Collaborative Innovation Center for Guangxi Ethnic Medicine, School of Chemistry and Pharmaceutical Sciences, Guangxi Normal University, Guilin, 541004 P.R. China; 2 College of Biotechnology, Guilin Medical University, Guilin, 541199 P.R. China

**Keywords:** Immunosuppression, Immunoediting

## Abstract

Latexin (LXN) plays an important role in tumorigenesis and inflammatory response and as a tumor suppressor in many tumors. However, whether LXN regulates tumorigenesis through immune regulation remains uncertain. Here, we demonstrate that *LXN* deficiency increases hematopoietic stem cells, as well as affects the proportion of immune cells in the peripheral system. Animal studies show that mice loss of *LXN* promotes tumor growth in subcutaneous tumor model and AOM/DSS-induced colorectal cancer model. We found that loss of *LXN* promotes macrophage M2 polarization and PD-L2 expression in macrophage, thus, inhibits the function of T cells. Adoptive transfer of wild-type macrophage rescues the function of T cells in *LXN*-deficient mice. *LXN* deficiency in hematopoietic lineage exacerbates colorectal carcinogenesis, and targeted inhibition of PD-L2 ameliorates cancer growth in *LXN*-deficient mice. Mechanistically, we demonstrate that LXN inhibits STAT3 transcriptional activity by targeting inhibition of JAK1 in macrophages. *LXN* deficiency enhances PD-L2 expression rather than PD-L1 in macrophages, which lead to inhibition of T cells in tumor microenvironment. Collectively, we define a critical role of LXN/JAK1/STAT3 signal in macrophage and highlights the potential role of LXN in tumor immune-escape by regulating macrophage polarization, as well as the expression of immune checkpoint PD-L2.

## Introduction

It is widely accepted that the immune system plays a dual role in tumor evolution, underlying tumor progression could be inhibited in a process called immunosurveillance or be promoted through immunosuppression [1–3]. Tumor microenvironment (TME) plays the key role in tumor immunomodulation, among the numerous cells in TME, tumor-associated macrophages (TAMs) and T cells are widely concerned [4–6]. TAMs mostly promote tumor growth, and high TAM content is usually associated with poor prognosis [7]. On the contrary, increased numbers of T cells, specifically activated cytotoxic T cells, are correlated with better survival in some cancers [8, 9]. Correspondingly, deficiencies in the number or functionality of immune cells in TME, such as CD8+ cytotoxic T cells, CD4+ helper cells, natural killer cells or B cells lead to increased susceptibility to carcinogen-induced tumors and spontaneous tumor development [10–12]. In this regard, it become a hot spot in oncology research that how do tumors develop into immune evasion and effectively restore immune surveillance.

Immune checkpoints are critical regulators of the immune system in TME, which regulate the duration and amplitude of the immune response to maintain self-tolerance and prevent autoimmunity [13–15]. For example, the checkpoint blockade therapy targeting programmed cell death 1 (PD-1) pathway has achieved unprecedented clinical effects in a wide range of tumors [16, 17]. The PD-1 pathway mainly contains two ligands, namely programmed death ligand-1 (PD-L1) and programmed death ligand-2 (PD-L2). Activated T cells expressing PD-1 may diminish their effector functions when PD-L1 or PD-L2 forms complex with PD-1 on cell surface, which leads to T cell-mediated immune escape by tumors [11, 18, 19]. The blockade of PD-1/PD-L1 pathway with anti-PD-1 or anti-PD-L1 antibodies successfully reinvigorate T cell functions and provide a durable response in different malignancies [12, 20]. Although immune checkpoint blockade (e.g., anti-CTLA4, anti-PD-1 or anti-PD-L1 treatment) has shown promising results in the clinic, usually only a fraction of patients respond to the treatment [16, 17, 21], suggesting other molecules, such as PD-L2, may also be important in predicting patient responses. However, the expression of PD-L2 in tumor tissue and its correlation with response to PD-1 axis targeted therapy has been less well-studied than PD-L1. Recently, much attention has been paid to the role of PD-L2 in tumor immunity. Yearley et al. reported that PD-L2 expression is observed in seven different tumor types (including renal cell carcinoma, bladder carcinoma, melanoma, non-small cell lung cancer, HNSCC, triple-negative breast cancer, and gastric carcinoma), especially in the patient samples absence of PD-L1 [22]. Moreover, PD-L2 expression is independently associated with clinical response in pembrolizumab-treated patients, indicating that presence or absence of PD-L2 expression may also play a role in response to PD-1 axis targeted therapies [22, 23].

Latexin (LXN) is the only known mammalian carboxypeptidase inhibitor, which is first identified in the lateral neocortex of rats [24, 25]. We and other groups have reported that LXN plays important roles in the inhibition of stem cell proliferation and tumor growth [26–28]. Recently, several studies have shown that LXN is closely related to immune associated diseases [29–31], however, the underlying mechanism remains uncertain to a large extent. Here, we provide evidence that LXN is involved in tumor immunomodulation by regulating macrophage PD-L2 expression. We report that mice loss of *LXN* significantly promoted the growth of cancer cells in subcutaneous tumor models, and *LXN*-deficient mice were more susceptibility to develop AOM/DSS-induced colorectal cancer. RNA-seq analysis of bone marrow derived macrophages (BMDMs) showed that *LXN*-deficient BMDMs had enhanced PD-L2 expression and tendency to polarize into M2 phenotype, which contributes to the immune escape of cancer cells by attenuating the function of T cells in tumor microenvironment. Mechanistically, we demonstrate that LXN inhibited STAT3 transcriptional activity by targeting inhibition of JAK1 in macrophage and LXN loss promotes PD-L2 expression rather than PD-L1 by enhancing JAK1/STAT3 signaling pathway.

## Results

### Effect of *LXN* deficiency on hematopoietic stem cells in bone marrow and immune cells in peripheral blood

**Fig. 1:**
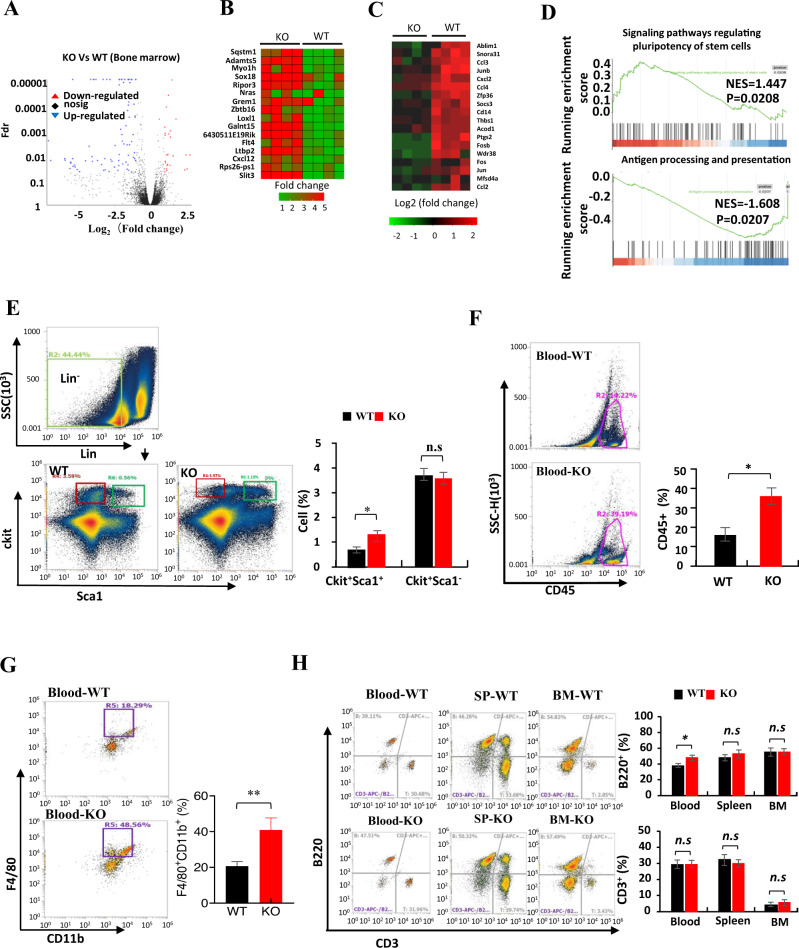
Effect of LXN deficiency on hematopoietic stem cells in bone marrow and immune cells in peripheral blood. **A** Volcano plot of gene expression changes of bone marrow from WT and LXN KO mice. **B**, **C** Heatmap of RNA-seq data showing the up- (**B**) and downregulation (**C**) genes in LXN deficiency bone marrow. **D** GSEA of RNA-seq data from the bone marrow of LXN KO versus WT mice using the signaling pathways regulating stem cell pluripotent, pathways in antigen processing and presentation gene set annotated in the KEGG. NES, normalized enrichment score. **E** Representative FACS plots and frequencies of Lin^−^Sca1^+^ckit^+^ in bone marrow from WT and LXN KO mice. **F**, **G** Representative FACS plots and frequencies of CD45^+^ (**F**), F4/80^+^CD11b^+^ (**G**) cells in peripheral blood from WT and KO mice. **H** Representative FACS plots and frequencies of B220^+^ and CD3^+^ cells in peripheral blood, spleen and bone marrow from WT and LXN KO mice. *n* = 6, *P < 0.05, **P < 0.01, n.s. no significance.

To begin to understand the effect of *LXN* knockout on immune system, we sequenced the transcriptome of *WT* and *LXN*^−/−^ (KO) bone marrow (BM) by RNA-seq assay and analyzed the distribution of immune cells in different tissues by FACS assay. Our RNA-seq data showed that *LXN* deficiency induced a significant upregulation of 25 genes expression, including the genes *Sqstm1*, *Sox18*, *Grem1* and *Slit3*, and downregulation of 86 genes, such as *Socs3*, *Thbs1*, *Fos* and *Jun* (Fig. 1A–C). GSEA analysis showed that the genes in signaling pathways regulating stem cell pluripotent were enriched in *LXN*^−/−^BM, while genes involved in antigen processing and presentation were decreased (Fig. 1D). As expected, FACS analysis showed that the proportion of Lin^−^Sca1^+^ckit^+^ (LSK) cells in *LXN* deficient BM increased significantly compared with wild-type mice (Fig. 1E), which is consistent with the report of Liu et al. [32], indicating the likelihood of increased self-renewal of *LXN*^−/−^ hematopoietic stem cells (HSCs). In peripheral blood, we observed that the proportion of CD45^+^ cells, F4/80^+^CD11b^+^ cells and B220^+^ cells increased in *LXN*^−/−^ mice, while the proportion of T cells did not change (Fig. 1F–H). There was no significant change in the proportion of B220^+^ cells and CD3^+^ cells in WT- and *LXN*^*−/−*^-BM and spleen (Fig. 1F). Collectively, these data demonstrated that *LXN* deficiency not only increased hematopoietic stem cells, but also affected the distribution of immune cells in the peripheral system, at least in part.

### Mice loss of *LXN* promotes the growth of cancer cells in the subcutaneous tumor model

**Fig. 2:**
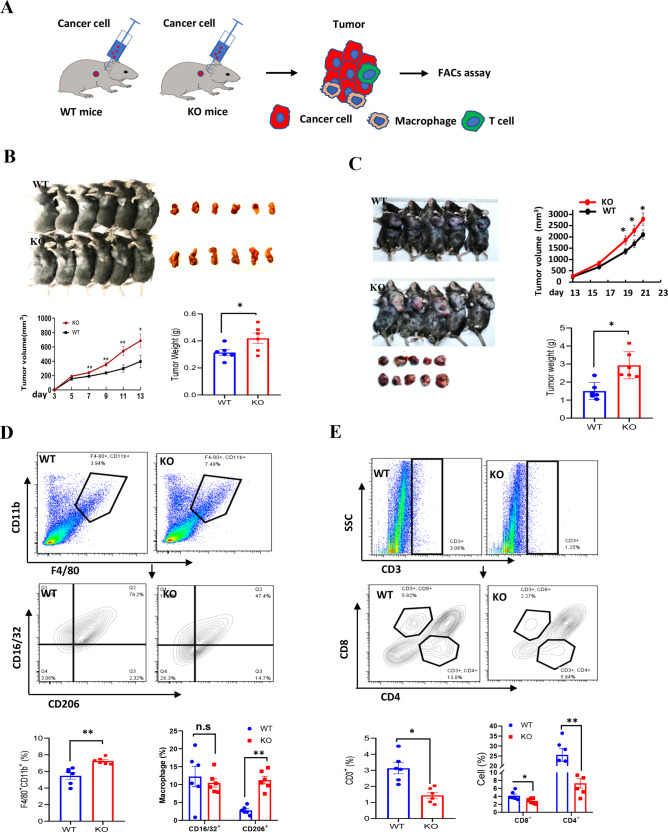
Loss of *LXN* in mice significantly promotes the growth of cancer cells by remodeling tumor microenvironment in the subcutaneous tumor models. **A** Experimental scheme for subcutaneous tumor assay. **B**, **C** MC38 cells (**B**) and LLC cells (**C**) were inoculated into the armpits of *WT* and LXN KO mice. The growth of the implanted tumor size was measured every 3 days and the tumor weight was measured at the end point (*n* = 6 mice). **D** Flow cytometry analysis of macrophages (F4/80^+^CD11b^+^) and the different subsets (M1, CD16/32^+^ CD206^−^; M2, CD16/32^−^CD206^+^) in tumor from WT and LXN KO mice. **E** Flow cytometry analysis of T cells (CD3^+^B220^−^) and the different subsets (CD8^+^T cell and CD4^+^T cell) in tumor from WT and LXN KO mice. Data are representative of three independent experiments. *P < 0.05, **P < 0.01, n.s. no significance.

To evaluate the effect of *LXN*-deletion on mice immune system, we examined the cancer cell growth in littermate wild-type (*WT*) and *LXN*^*−/−*^ mice (KO) by subcutaneous tumor model (Fig. 2A). Cancer cells were inoculated into the armpits of *WT* and *LXN*^*−/−*^ mice. The results showed that mice loss of *LXN* significantly promoted the growth of cancer cells over the entire assay period in subcutaneous tumor model, no matter loaded with colorectal cancer or lung cancer cells (Fig. 2B, C). FACs assay showed that the proportion of F4/80^+^CD11b^+^CD16/32^−^CD206^+^ macrophage (M2-macrophage) was remarkably increased in the implanted tumors of *LXN*^*−/−*^ mice (Fig. 2D), while T cell (CD3^+^CD4^-^CD8^+^ and CD3^+^CD8^-^CD4^+^) decreased significantly (Fig. 2E). Collectively, our data preliminarily demonstrated that *LXN* deficiency remodeled the pro-tumor microenvironment of mice by promoting the infiltration of M2-macrophages and attenuating T cells in tumor tissue, thus promoting the growth of subcutaneous tumor.

### *LXN*-deficient macrophage inhibits the function of T cells in vitro

**Fig. 3:**
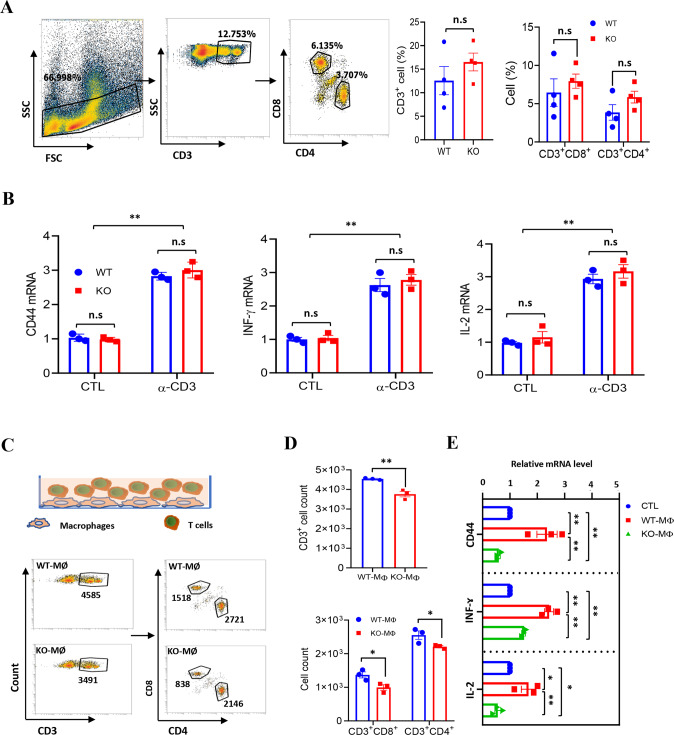
*LXN*-deficient macrophages inhibit T cell activity in vitro. **A** Flow cytometry analysis of T cells isolated from spleen of WT and LXN KO mice. Representative FACS plots (left) and frequencies (right) of T cells. *n* = 4, n.s. no significance. **B** T cells from WT and LXN KO mice were activated by CD3 (5 μg/mL) antibodies for 48 h. The activity of T cells was evaluated by measuring the expression of *CD44, IFN-γ* and *IL-2*, by qRT-PCR. *n* = 3, **P < 0.01, n.s. no significance. **C**–**E** Isolated T cells (1 ×;10^4^ per well) were co-cultured in 96-well plates with a 2:1 ratio of the WT or LXN KO macrophage for 72 h. T cells were analyzed by flow cytometer (**C**), the count of CD3^+^, CD3^+^CD4^+^ and CD3^+^CD8^+^ cells are showed (**D**). The production of *CD44*, *INF-ɤ* and *IL-2* were determined by qRT-PCR (**E**). *n* = 3, *P < 0.05, **P < 0.01, ***P < 0.001, n.s. no significance. Data are representative of three independent experiments.

Since it was observed that *LXN* deficiency would increase macrophages and reduce the infiltration of T cells in subcutaneous tumors, we asked whether *LXN* deletion would change the function of immune cells (such as macrophages and T cells) in vitro. We first evaluated the impact of *LXN* deficiency on T cells by analyzing the content of T cells in spleen. We found that *LXN* knockout had no significant effect on CD3^+^, CD3^+^CD8^+^ and CD3^+^CD4^+^ T cells in spleen (Fig. 3A). We also measured cytokines to characterize the activity of *WT* and *LXN*^−/−^ T cells. Our data showed that *LXN* knockout had no effect on T cell activation under basal or anti-CD3e antibody (α-CD3e) treatment (Fig. 3B). We then FACS-sorted BMDMs (F4/80^+^CD11b^+^) from *WT* and *LXN*^−/−^ mice and labeled with CFSE. Next, we co-cultured CFSE-labeled *WT* or *LXN*^−/−^ BMDMs (WT-MØ or KO-MØ) with T cell (Fig. 3C). FACS assay show that CFSE^-^CD3^+^ cells, CFSE^-^CD3^+^CD4^+^ cells and CFSE^−^CD3^+^CD8^+^ cells decreased when co-cultured with KO-MØ (Fig. 3D). QPCR showed that the level of *CD44*, *INFɤ* and *IL-2* (Markers of T cell activation) were much higher in WT-MØ-induced T cells than that of KO-MØ-induced (Fig. 3E), suggesting that T cells co-cultured with *LXN*^*−/−*^ macrophages developed dysfunction, and existed in immunosuppressive state.

### *LXN* deficiency enhances PD-L2 expression in macrophages and promotes M2 phenotype polarization

**Fig. 4:**
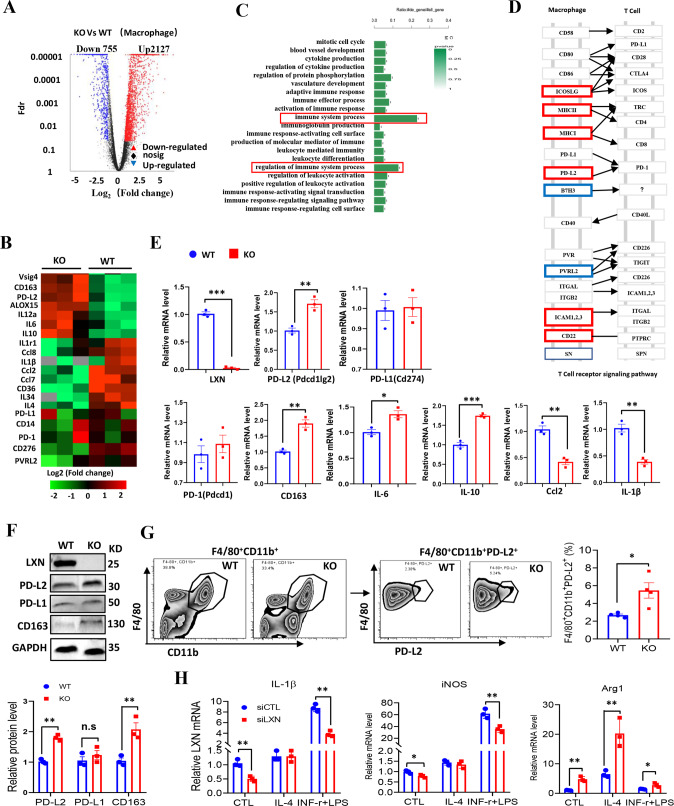
Loss of *LXN* upregulates PD-L2 expression in macrophage and increases polarization towards M2 phenotype. **A** BMDMs from WT and LXN KO mice were subjected to RNA-seq. Volcano plot of gene expression changes in LXN KO and WT macrophages. **B** Heatmap of RNA-seq data showing the up- and downregulation genes in *LXN*-deficient macrophages (*n* = 3 mice). **C** GO analysis of upregulated and downregulated genes suggested the enrichment in the regulation of immune system. **D** Representative KEGG analysis of macrophage adhesion molecules in T cell receptor signaling pathway. **E** Relative expression levels of cytokine and chemokine mRNA in WT and LXN KO macrophage were measured by qRT-PCR. *n* = 3, *P < 0.05, **P < 0.01, ***P < 0.001. **F** Western blot showed *LXN* deficiency upregulates the expression PD-L2, PD-L1 and CD163 in macrophages. **P < 0.01, n.s. no significance. **G** Representative FACS plots and frequencies of PD-L2^+^ populations in F4/80^+^CD11b^+^ cells from *WT* or *LXN*-deficient mice. *n* = 4, ^*^P < 0.05. **H** Representative qRT-PCR analysis of M1 (IL-1β, iNOS) and M2 (Arg1) macrophage markers in RAW264.7 cells transfected with CTL or *LXN* siRNA after induction of IL-4 (20 ng/ml) or IFN-ɤ+LPS (30 ng/ml+100 ng/ml) for 72 h. *n* = 3, *P < 0.05, **P < 0.01. *n* = 3, **P < 0.01. Data are representative of three independent experiments.

To understand how *LXN* deficiency affects macrophage function, the gene profiling of bone marrow derived macrophages (BMDMs) from *WT* and *LXN*^−/−^ mice was performed by RNA-seq assays. Our data revealed that *LXN* deficiency induced a significant upregulation of 2127 genes, including *Vsig4*, *ALOX15, PD-L2, CD163*, and *IL-10*, and downregulation of 755 genes, such as *CD276*, *IL-1β* and *Ccl2* (Fig. 4A, B). Gene ontology analysis demonstrated that the genes involved in the regulation of immune system process were most frequently regulated (Fig. 4C, D). The upregulation of *PD-L2*, *CD163* and *IL-10* and downregulation of *Ccl2* and *IL-1β* in *LXN* deficiency BMDMs were confirmed by qPCR (Fig. 4E). The expression of PD-L2 and CD163 in *LXN*^*−/−*^ BMDMs were further confirmed by Western blot (Fig. 4F). The expression of PD-L2 in macrophage also be confirmed by FACs. We found that the F4/80^+^CD11b^+^ macrophage from the *LXN*^−/−^ mice expressed higher level of PD-L2 (F4/80^+^CD11b^+^PD-L2^+^) than that from *WT* mice (Fig. 4G).

Since the increased expression of genes related to M2-macrophage phenotype (such as *Vsig4*, *ALOX15* and *CD163*) after *LXN* deletion, we also investigated whether LXN regulates macrophage polarization. RAW 264.7 cells were stimulated with a combination of IFN-ɤ and LPS to induce M1- or IL-4 to induce M2-macrophage polarization. We found that cells lacking *LXN* showed a significant increase in the tendency to polarize towards M2 phenotype, as monitored by *Arg1* mRNA expression, but a decrease in the tendency to polarize towards M1 phenotype, as monitored by expression of *iNOS* and *IL-1β* mRNA (Fig. 4H). Collectively, our data demonstrated that *LXN* deficiency in macrophage promoted PD-L2 expression and increased the tendency to polarize towards M2 phenotype, suggesting *LXN*-deficient macrophages have a potential immunosuppressive phenotype.

### *LXN* deficiency promotes PD-L2 expression rather than PD-L1 by enhancing JAK1/STAT3 signaling pathway in macrophages

**Fig. 5:**
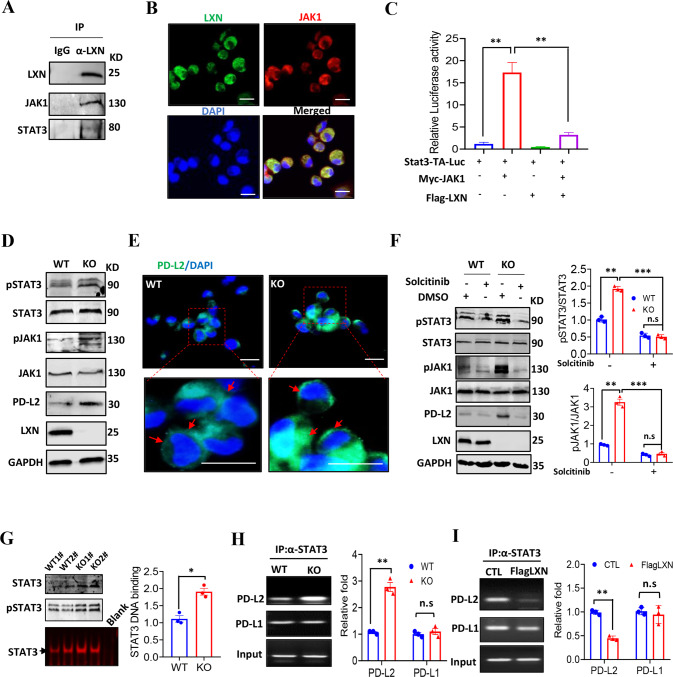
Macrophage deficiency of *LXN* promotes PD-L2 expression by enhancing JAK1/STAT3 activity. **A** Immunoblotting shows that LXN interacts with JAK1 in BMDMs. **B** Representative co-localization of LXN and JAK1 in BMDMs by immunofluorescence. Scale bars = 20 µm. **C** RAW264.7 cells were transfected with pStat3-TA-luc, Myc-JAK1 and Flag-LXN plasmid as indicated, and induction of luciferase activity was determined. Data are representative of three independent experiments. **P < 0.01. **D** Western blot shows that *LXN* deficiency increases the expression of PD-L2 and phosphorylation of JAK1/STAT3 in mice macrophages. **E** Representative the expression of PD-L2 in macrophages from WT and LXN KO mice by immunofluorescence. The red arrow indicates that PD-L2 is expressed on the cell surface. Scale bars = 20 µm. **F** Macrophages from WT and LXN KO mice treated with solcitinib for 24 h, the expression of PD-L2 and the activity of JAK1/STAT3 were determined by Western blot (left). Quantitative analysis of western blot was shown (right). Data are representative of three independent experiments. **P < 0.01, ***P < 0.001, n.s. no significance. **G** Representative the DNA-binding activity of STAT3 in WT and LXN KO macrophage by EMSA assay. *P < 0.05. **H** Representative the binding activity of STAT3 to PD-L1/2 promoter in WT and LXN KO macrophages by ChIP assay. **I** Representative the binding activity of STAT3 to PD-L1/2 promoter in RAW 264.7 cells transfected with Flag LXN. Data are representative of three independent experiments. **P < 0.01, n.s. no significance.

To better understand the molecule mechanism(s) underline LXN regulating macrophage, co-immunoprecipitation was performed. We found that LXN, JAK1 and STAT3 form a complex in BMDMs (Fig. 5A). The interaction of LXN and JAK1 was further confirmed in BMDMs by confocal (Fig. 5B). We performed STAT3 binding sites-driven luciferase (Stat3-TA-Luc) assay in RAW264.7 cells, and found that overexpression of JAK1 substantially increase the Stat3-TA-luc activity, which was markedly inhibited when JAK1 and LXN were co-expressed (Fig. 5C), indicating that LXN inhibited STAT3 transcriptional activity by targeting JAK1. JAK/STAT has been reported to involved in the regulation of PD-L1/L2 expression [33]. Our results showed that *LXN* deletion in macrophage increased expression of PD-L2, however, had no effect on the expression of PD-1 and PD-L1 (Fig. 4E, F). We speculated that this may be related to the specific activation of STAT3 in *LXN*-deficient macrophages. We demonstrated that *LXN* deficiency in BMDMs significantly increased the phosphorylation of JAK1 and STAT3, as well as the expression of PD-L2 (Fig. 5D, E). In addition, the phosphorylation of JAK1 and STAT3 and the expression of PD-L2 caused by *LXN* deficiency could be reversed by the treatment of macrophages with JAK1 inhibitor (Fig. 5F). These results indicated that LXN was a suppressor of JAK1/STAT3 activation in macrophages. When LXN did not exist, the inhibitory effect of JAK1 was released, thereby promoting STAT3 activity and PD-L2 expression. Therefore, our results further support the key role of LXN and JAK1 contribution to the activity of STAT3 in macrophages.

STAT3 regulates many inflammatory- and immune-related genes [34, 35]. To further characterize the transcriptional activity of STAT3 in macrophage after *LXN* knockout, EMSA and ChIP assay were performed. The results showed that *LXN* deficiency enhanced the DNA binding activity of STAT3 in BMDMs (Fig. 5G), and extremely increased the binding of STAT3 to *PD-L2* promoter, but no significantly change of binding to the *PD-L1* promoter (Fig. 5H). In contrast, overexpression of LXN attenuated the binding of STAT3 to *PD-L2* promoter (Fig. 5I). Taken together, these findings collectively indicated that LXN interacted with JAK1 and inhibited the activation of JAK1/STAT3 signaling, thus negatively regulating the expression of PD-L2 in macrophages.

### *LXN* deficiency remodels immune microenvironment by increasing PD-L2^+^ macrophages and reducing T cells infiltration in colorectal cancer tissues in vivo

**Fig. 6:**
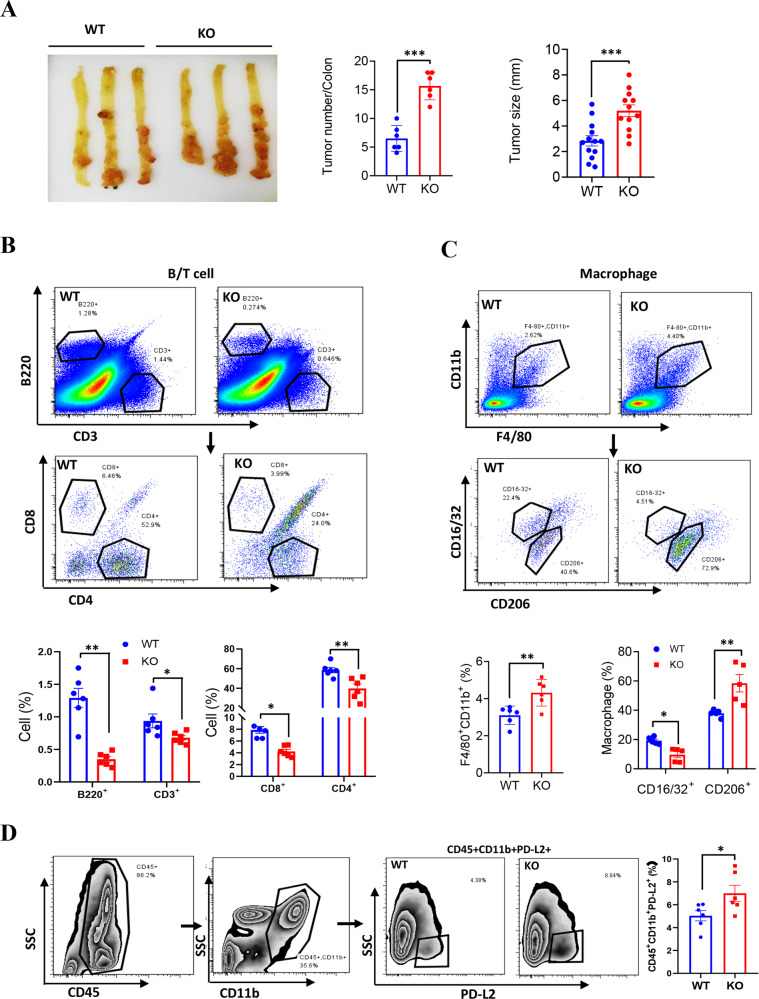
*LXN* deficiency increases enhances CD45^+^CD11b^+^PD-L2^+^ macrophages in AOM/DSS-induced colonic tumor tissues. **A** Gross view of colonic tumor of AOM/DSS-treated WT and LXN KO mice. Tumor number and size were counted (*n* = 6 mice). **P < 0.01. **B** Flow cytometry analysis of B cell (CD3^−^B220^+^), T cells (CD3^+^B220^−^) and the different subsets (CD8^+^T cells and CD4^+^T cells) in colon tissue in WT and LXN KO mice treated with AOM/DSS. **C**, **D** Flow cytometry analysis of macrophages (F4/80^+^CD11b^+^), the different subsets (M1, CD16/32^+^CD206^−^; M2^,^ CD16/32^−^CD206^+^) (**C**) and CD45^+^**C**D11b^+^PD-L2^+^ cells (**D**) in colon tissue in WT and LXN KO mice treated with AOM/DSS. *n* = 6, *P < 0.05, **P < 0.01. Data are representative of three independent experiments.

To determine whether PD-L2^+^ macrophages increased in tumor microenvironment in *LXN*-deletion mice, we first generated AOM/DSS-induced colorectal cancer model in WT and *LXN*^*−/−*^ mice (Supplementary Fig. S1A). We showed that *LXN*^*−/−*^ mice developed more severe clinical symptoms, including rapid weight loss, a lower survival rate, a higher disease activity index (DIA) and bloody stool, shortened colon and more spleen gain compared with WT mice (Supplementary Fig. S1B–G). Tumors occurred more frequently, and tumor loads were heavier in *LXN*^*−/−*^ mice than in WT mice (Fig. 6A). These results demonstrated that *LXN*-deficient mice were more susceptible to AOM/DSS-induced colorectal tumorigenesis in vivo. Then, primary colorectal tumor tissues were isolated from AOM/DSS-treated mice and analyzed by FACs assay. We found that B220^+^ cell and CD3^+^ T cell were significantly reduced in the colorectal tumor of *LXN*^−/−^ mice compared to WT mice (Fig. 6B). However, F4/80^+^CD11b^+^ macrophages, particularly PD-L2^+^ macrophages (CD45^+^CD11b^+^PD-L2^+^) infiltrated in the colorectal tumor of *LXN*^−/−^ mice was significantly higher than that in *WT* mice (Fig. 6C, D). Further analysis of subpopulations showed that F4/80^+^CD11b^+^CD16/32^−^CD206^+^ cell (M2-macrophage) was significantly increased; however, F4/80^+^CD11b^+^CD206^−^CD16/32^+^ cell (M1-macrophage), CD3^+^CD4^−^CD8^+^ (CD8^+^T cell) and CD3^+^CD8^-^CD4^+^ (CD4^+^T cell) cells were decreased in *LXN*^*−/−*^ mice compared to that of *WT* mice (Fig. 6C, D). Collectively, these data implicated that *LXN* deficiency increased PD-L2^+^ macrophages and decreased T cells infiltration in colorectal cancer tissues, suggesting *LXN* deficiency enhances pro-tumor immune response in mice.

### *LXN* deficiency in hematopoietic lineage exacerbates tumorigenesis in vivo

**Fig. 7:**
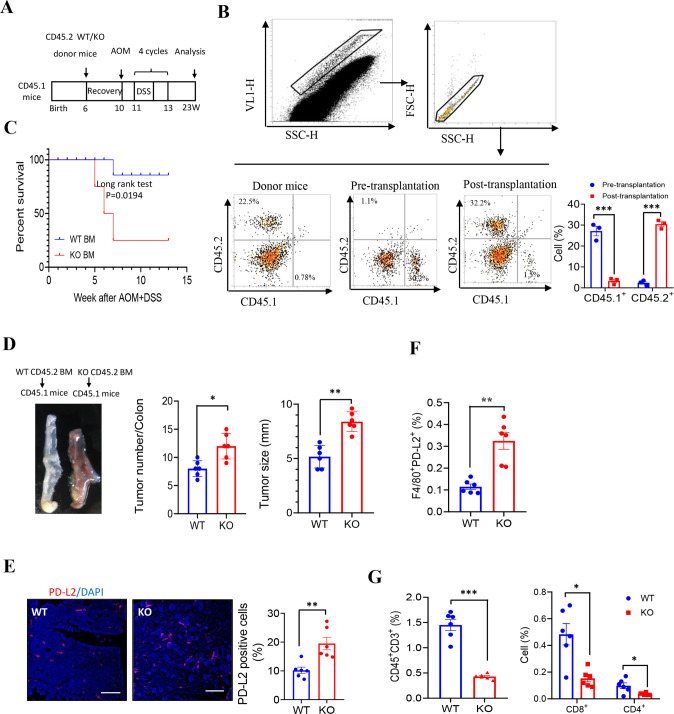
*LXN* deficiency in hematopoietic lineage exacerbates tumorigenesis in vivo. **A** Schematic representation of mouse model of bone marrow transplantation. Bone marrow transfers were performed in 6-weeks old lethally irradiated *WT* mice (CD45.1 background) followed by reconstitution with bone marrow from WT or LXN KO mice (CD45.2 background), respectively. Then, four cycles of AOM/DSS treatment to induce colorectal cancer. **B** Representative FACS plots show CD45.2^+^ and CD45.1^+^ cells in the peripheral blood of donor mice and recipient mice before and after bone marrow transplantation, respectively. **C** Survival of AOM/DSS-treated mice (CD45.1 background) after conducted transplantation with WT and LXN KO bone marrow (CD45.2 background) (*n* = 18 mice per group). **D** Representative images of colorectal tissue of each group chimeras and tumor number and size were counted. *n* = 6, **P < 0.01, ***P < 0.001. **E** Representative images of cross-sections of the tumor tissues from each group stained with anti-PD-L2 antibody. Quantification of PD-L2 positive cells are shown. Scale bars = 100 µm. *n* = 6, **P < 0.01. **F**, **G** Frequencies of WT or LXN KO donor (CD45.2)-derived F4/80^+^PD-L2^+^ cells (**F**), CD3^+^, CD3^+^CD8^+^ and CD3^+^CD4^+^ T cells (**G**) in the tumor of recipient mice (CD45.1) after 4 cycles of AOM/DSS treatment. *n* = 6, *P < 0.05, **P < 0.01, ***P < 0.001. Data are representative of three independent experiments.

To confirm the role of macrophage-derived *LXN* in tumor immunomodulation, bone marrow transplantation (BMT) was performed. Donor BM from *WT* or *LXN*^−/−^mice (CD45.2 background) was engrafted into lethally irradiated host mice (CD45.1 background). After 4-week recovery, mice were put on 4 cycles AOM/DSS induction (Fig. 7A). Flow cytometric analysis of CD45.2^+^ cells in blood to confirm the success of transplantation (Fig. 7B). After AOM/DSS treatment, mice received *LXN*^−/−^BM exhibit significantly shortened survival (Fig. 7C). Consistently, the tumor loads and tumor size were higher in mice received *LXN*^−/−^BM than in mice received *WT* BM (Fig. 7D). We also observed a significantly increased of PD-L2^+^ cells in the colorectal tumor tissues in AOM/DSS-induced mice received *LXN*^−/−^BM (Fig. 7E, F). As expected, T cell (CD45^+^CD3^+^), the subpopulation of CD8^+^ T cell (CD3^+^CD4^−^CD8^+^) and CD4^+^ T cell (CD3^+^CD8^−^CD4^+^) were decreased in mice transplanted with *LXN*^−/−^BM (Fig. 7G). These results of bone marrow chimeras strongly suggested that *LXN-*deficient hematopoietic lineage accelerated AOM/DSS-induced colorectal tumorigenesis in mice.

### Adoptive therapy with WT macrophages rescues the function of T cells in *LXN*-deficient mice

**Fig. 8:**
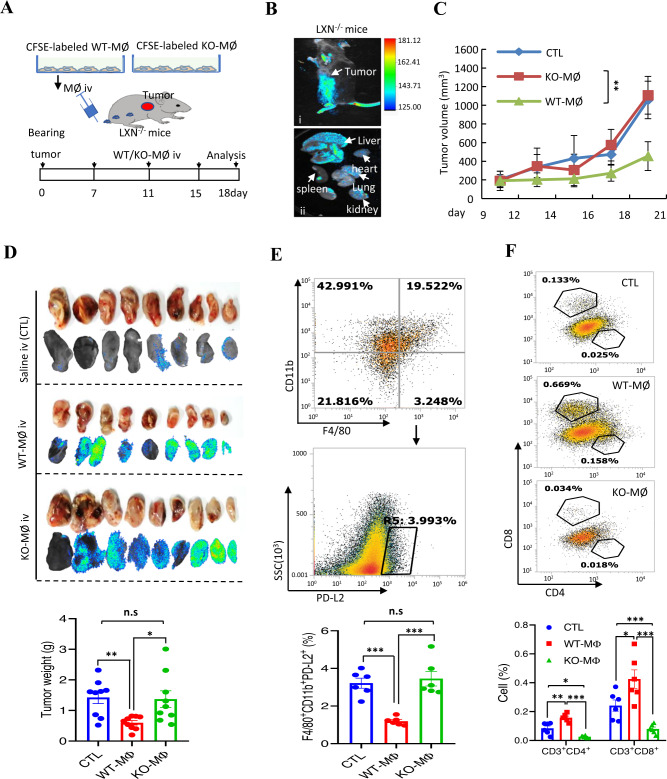
Adoptive therapy with WT macrophages rescues the function of T cells in *LXN*-deficient mice. **A** Schematic representation of the mouse model of cell adoptive therapy for in the subcutaneous tumor models. Macrophages isolated from bone marrow of WT and LXN KO mice were labeled with CFSE. MC38 cells were engrafted into the subcutaneously of LXN KO mice. The CFSE-labeled *WT* or LXN KO-macrophages were injected twice through the tail vein at the indicated time points. **B** Animal imaging shows the enrichment of CFSE-labeled macrophages at the tumor site (i) and other organs (ii), as shown by the white arrow. **C** Representative the tumor growth curves. *n* = 9, *P < 0.05, **P < 0.01, n.s. no significance. **D** Imaging of tumor tissues of tumor-bearing recipient LXN KO mice that were treated with CFSE-labeled WT or LXN KO-Macrophages, and the tumor weight at the end point was shown. *n* = 9. **E** Representative FACS plots and frequencies of F4/80^+^CD11b^+^PD-L2^+^ macrophages in subcutaneous tumors treated with WT or LXN KO-Macrophages. *n* = 6, ***P < 0.001, n.s. no significance. **M** Representative FACS plots and frequencies of CD3^+^CD4^+^ and CD3^+^CD8^+^ T cells in subcutaneous tumors treated with WT or LXN KO-Macrophages. *n* = 6, *P < 0.05, **P < 0.01, ***P < 0.001.

To further prove the inhibitory effect of *LXN*-deficient macrophages on tumor immunity, adoptive therapy with *WT* macrophages was performed on tumor bearing *LXN*^*−/−*^ mice. BMDMs from *WT* and *LXN*^−/−^ mice were labeled with CFSE. Cancer cells were engrafted into the subcutaneously of *LXN*^−/−^ mice. After 7 days, the CFSE-labeled *WT* or KO-MØ were injected through the tail vein at the indicated time points (Fig. 8A). Animal imaging show that CFSE-labeled macrophages were enriched in tumor sites (Fig. 8B-i), as well as enriched in the liver (Fig. 8B-ii). After 18 days, large number of CFSE-labeled macrophages were observed in tumor tissue, and the tumors of WT-MØ-treated group were significantly smaller than that of KO-MØ-treated group (Fig. 8C, D), indicating that KO-MØ possessed the characteristics of tumor associated macrophages (TAM). Compared with the KO-MØ injection group, fewer F4/80^+^CD11b^+^PD-L2^+^ macrophages could be detected in the WT-MØ injection group (Fig. 8E). Upon analyzing T cell infiltration in tumor tissue, we found that, as expected, WT-MØ injected into *LXN*^−/−^ mice increased CD3^+^CD4^+^ and CD3^+^CD8^+^ T cells in tumor tissue, however, KO-MØ further attenuated T cells (Fig. 8F), supporting the hypothesis that *LXN*-deficient macrophages inhibited T cells, while WT-MØ could rescue the function of T cells in *LXN*^*−/−*^ mice, at least in partial, in tumor microenvironment.

### PD-L2 blockading attenuates colorectal cancer cell growth in *LXN*-deficient mice

**Fig. 9:**
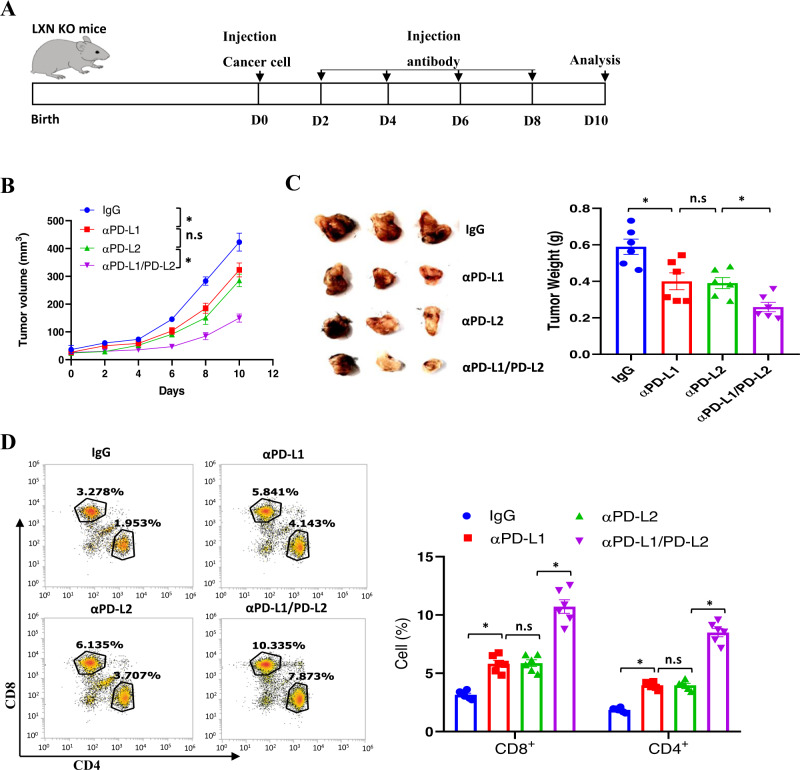
PD-L2 blockading therapy on colorectal cancer in *LXN*-deficient mice. **A** Experimental time line for the implantation of MC38 cells in LXN KO mice and therapeutic schedule. **B** Cancer cell bearing mice were treated with anti-PD-L1, anti-PD-L2, anti-PD-L2/PD-L2, or IgG control. The growth of the implanted tumor size was measured every 2 days. *n* = 6, *P < 0.05, n.s. no significance. **C** Representative images of mice bearing tumor formed by MC38 cells, and quantification of tumor weight at the end point after antibody therapy. *n* = 6, *P < 0.05, n.s. no significance. **D** Representative dot plots and of quantification of CD4+ and CD8+ T cells in tumor after antibody therapy. *n* = 6, *P < 0.05, n.s. no significance.

The PD-L1/2/PD-1 axis represents an immune-inhibiting checkpoint mediating tumor immune evasion [18, 33]. We observed the increased PD-L2 levels in *LXN*-deficient macrophage, we accordingly evaluated the effects of PD-L2 and PD-L1 blockade on cancer growth. To this end, MC38 cells were engrafted into the subcutaneously of *LXN*^*−/−*^ mice. After 2 days, these mice were injected intraperitoneally with a 20 mg/kg dose of monoclonal anti-PD-L1, anti-PD-L2, or anti-PD-L1/PD-L2 blocking antibody, IgG as control antibodies, every 2 days, for a period of 10 days, then tumor tissues were collected and determined (Fig. 9A). Anti-PD-L2-treated mice displayed significant reduction in the growth of tumor volume over the entire assay period and tumor weight at the end point in subcutaneous models compared with control antibody-treated mice, although there was no significant difference between the anti-PD-L1 and anti-PD-L2 treated groups (Fig. 9B). Notably, we observed that the combination of anti-PD-L1 and anti-PD-L2 has the best tumor therapeutic effect (Fig. 9B, C). Consistent with these results, our data showed that CD8^+^ and CD4^+^ T cells were significantly higher in anti-PD-L2 or anti-PD-L1/PD-L2 treated group compared with control group (Fig. 9D). Taken together, our data from antibody therapy further demonstrated that the increase of PD-L2 in *LXN*-deficient macrophage was critical for tumorigenesis.

## Discussion

The present study, for the first time, demonstrated a novel mechanism by which LXN regulated tumorigenesis and immune escape through JAK1/STAT3/PD-L2 pathway. We show that mice loss of *LXN* significantly promote the growth of cancer cells in subcutaneous tumor models, and mice are more susceptibility to develop AOM/DSS-induced colorectal cancer in vivo. *LXN*-deficient macrophages inhibit the function of T cells in vitro, and adoptive transfer of wild-type macrophages rescue the function of T cells in *LXN*-deficient mice. Bone marrow transplantation prove that *LXN*-deficient hematopoietic lineage accelerates AOM/DSS-induced colorectal tumorigenesis. Mechanistically, we prove that LXN interacts with JAK1, which inhibit the activation of STAT3 in macrophages. Loss of *LXN* in macrophage activates JAK1/STAT3/PD-L2 pathway, which contribute to the immune escape of cancer cells by attenuating the function of T cells in TME. Importantly, targeted blockade of PD-L2 attenuates tumorigenesis in *LXN* loss mice. Thus, these findings provide evidences that LXN functions as a negative regulator of tumorigenesis and tumor immune escape, which expand the understanding of the antitumor effect of LXN previously unknown and provide a molecular mechanistic rationale for combining anti-PD-L2 immune-checkpoint therapy of tumor patients with low expression of LXN.

Since Liang et al. revealed the negative regulatory function of LXN on the proliferation of mouse hematopoietic stem cells, many researchers have speculated that LXN may play a role in the immune system [26, 27, 36]. For example, Heinrich et al. reported *LXN* mRNA was significantly higher in M1-macrophages as compared to M0- and M2-macrophages, suggesting that LXN might be a biomarker of M1-macrophages, although they did not ultimately prove this hypothesis due to the limitations of the detection method [37]. Seed et al. reported that LXN could be secreted by prostate epithelial cells, and prospectively proposed that LXN might change immune microenvironment by acting on B cells and T cells. Unfortunately, they did not provide further data to support the hypothesis [38]. To evaluate the impact of LXN on immune system, we analyzed the changes of immune cells in bone marrow and peripheral blood in mice after *LXN* knockout. Our results show that *LXN* deficiency not only increase hematopoietic stem cells, but also affect the distribution of immune cells in the peripheral system (Fig. 1). We further tested the tolerance of *WT* and *LXN*^*−/−*^ mice to tumor growth in subcutaneous tumor models. We found that cancer cells were easier to grow in *LXN*-deficient mice, and more M2 macrophages (F4/80^+^CD11b^+^CD16/32^−^CD206^+^) and fewer T cells (CD3^+^CD4^+^ and CD3^+^CD8^+^) were observed in the tumor tissues of *LXN*^*−/−*^ mice (Fig. 2), suggesting it is universal that the changes of immune environment caused by *LXN* deficiency are conducive to tumor growth. These data strongly support our hypothesis that LXN indeed affect tumor cell growth through immune regulation. RNA-seq further support the hypothesis that *LXN* deficiency in macrophage increase the tendency to polarize towards M2 phenotype (Fig. 4), because some genes related to M2 increased in *LXN*^−/−^ macrophages, including *CD163*, *ALOX15, IL-10,* and *Vsig4* [39–41]. Conversely, the expression of M1-related genes such as *CCL2* and *IL-1β* decreased. Indeed, several studies have indicated that the STAT3 pathway mediates M1/M2 macrophage polarization during the development of cancer and other diseases [42, 43]. Moreover, it was reported that myeloid cell-specific disruption of suppressor of cytokine signaling 3 (SOCS3), the negative regulator of STAT3, makes macrophage differentiate toward an M2 phenotype [44]. Interestingly, Akiyama et al. reported that LXN expression was upregulated in many cells after STAT3 knockdown, indicating the association between LXN and STAT3 pathway [45]. Together, these findings support our hypothesis that *LXN* knockout has a negative impact on the immune system of mice, at least in partial to promote the polarization of macrophages to M2 phenotype by activating JAK/STAT3 pathway.

As evidence of LXN regulating the TME, we excited to find that *LXN* deficiency significantly promotes the expression of immune-checkpoint molecule PD-L2 in macrophage (F4/80^+^CD11b^+^PD-L2^+^) by activating STAT3 (Figs. 4 and 5). PD-L2 is the second ligand of PD-1, which inhibits T cell activation and participates in immunosuppression [18, 33]. Messal et al. found that tumor microenvironment could induce expression of PD-L2 in immune cells [46]. Interestingly, Jennifer et al. analyzed more than 400 tumor samples and found that PD-L2 was observed in many types of cancer [22]. They found that patients with both positive PD-L1 and PD-L2 were more effective than those with positive PD-L1, and the expression of PD-L2 was independent in predicting the clinical efficacy of keytruda in patients, they thus speculated the expression of PD-L2 may provide more information than PD-L1 in predicting the clinical efficacy of anti-PD-1 drugs [22]. Interestingly, Wang et al. demonstrated that PD-L2 was expressed in approximately 40% colorectal cancer (CRCs), however, PD-L1 expression in only 12% patients, and it was PD-L2 but not PD-L1 expression independently associated with poor survival of CRC patients, further suggesting the special role of PD-L2 in tumor immunity [47]. In this work, we found that macrophages loss of *LXN* promote the level of p-STAT3 and *PD-L2* rather than *PD-L1* (Fig. 5). *PD-L1* and *PD-L2* are paralog genes, and their promoters share a similar architecture in terms of putative binding sites (i.e., the binding sites of *PD-L1* promoter include STAT1/3, STAT2/5 and IRF1; the binding sites of *PD-L2* promoter include STAT1/3, IRF1α and IRF1β). Interestingly, IRF1 is the key factor of *PD-L1* promoter function, while *PD-L2* is mainly regulated by STAT1/3 [33], which also explain our results that only PD-L2 is upregulated in *LXN*^*−/−*^ macrophages. It has been reported that PD-1 ligands (PD-Ls) contribute to the M2 polarization and immunosuppressive effects of macrophages [48], and PD-L2 expression correlated with other established markers for alternatively activated macrophages [49]. As early as ten years ago, Huber et al. reported that PD-L2 appears to be a more specific and distinctive marker for M2, and IL-4 induced the expression of PD-L2 on classically activated macrophages, however, LPS increased the expression of PD-L1 on M2 [50]. In this regard, it can be concluded that PD-L2 plays a role in the anti-inflammatory reprogramming of macrophages. Our in vitro and in vivo data clearly showed the increase of PD-L2^+^ macrophage after *LXN* deletion. More importantly, the importance of the upregulation of PD-L2 in *LXN*^*−/−*^ macrophage in tumor immune escape is highlighted by bone marrow transplantation (Fig. 7), adoptive therapy (Fig. 8) and immune checkpoint suppression therapy by targeting blockade of PD-L2 (Fig. 9). These data provide evidence for the effectiveness of anti-PD-L2, especially in combination with anti-PD-L1 therapy in cancer patients with low expression of LXN.

In conclusion, the present study revealed the crucial role of LXN in tumor tumorigenesis from an immunological perspective, and its ability to regulate tumor microenvironment through modulating JAK1/STAT3/PD-L2 axis in macrophages. These findings greatly expand our understanding of LXN as a tumor suppressor and highlight the potential role of LXN in tumor immunomodulation by regulating the polarization of macrophage as well as the expression of immune checkpoint molecule PD-L2, which we believe has practical significance in the clinical treatment of immune-checkpoint of tumor patients.

## Materials and methods

Materials and methods are available in the online-only Data Supplement.

## Supplementary information

Supplementary material

Supplementary Figures

Supplementary Table S1

Supplementary Table S2

Supplementary Table S3

Original Data File

## Data Availability

The data that support the findings of this study are available from the corresponding authors upon reasonable request.

## References

[1] Dunn GP, Bruce AT, Ikeda H, Old LJ, Schreiber RD (2002). Cancer immunoediting: from immunosurveillance to tumor escape. Nat Immunol.

[2] Grivennikov SI, Greten FR, Karin M (2010). Immunity, inflammation, and cancer. Cell.

[3] Drake CG, Jaffee E, Pardoll DM (2006). Mechanisms of immune evasion by tumors. Adv Immunol.

[4] Yunna C, Mengru H, Lei W, Weidong C (2020). Macrophage M1/M2 polarization. Eur J Pharmacol.

[5] Sica A, Mantovani A (2012). Macrophage plasticity and polarization: in vivo veritas. J Clin Investig.

[6] Funes SC, Rios M, Escobar-Vera J, Kalergis AM (2018). Implications of macrophage polarization in autoimmunity. Immunology.

[7] Murdoch C, Muthana M, Coffelt SB, Lewis CE (2008). The role of myeloid cells in the promotion of tumour angiogenesis. Nat Rev Cancer.

[8] Galon J, Costes A, Sanchez-Cabo F, Kirilovsky A, Mlecnik B, Lagorce-Pages C (2006). Type, density, and location of immune cells within human colorectal tumors predict clinical outcome. Science.

[9] Swann JB, Smyth MJ (2007). Immune surveillance of tumors. J Clin Investig.

[10] Meng X, Liu X, Guo X, Jiang S, Chen T, Hu Z (2018). FBXO38 mediates PD-1 ubiquitination and regulates anti-tumour immunity of T cells. Nature.

[11] Latchman Y, Wood CR, Chernova T, Chaudhary D, Borde M, Chernova I (2001). PD-L2 is a second ligand for PD-1 and inhibits T cell activation. Nat Immunol.

[12] Tan CL, Kuchroo JR, Sage PT, Liang D, Francisco LM, Buck J (2021). PD-1 restraint of regulatory T cell suppressive activity is critical for immune tolerance. J Exp Med.

[13] Paluch C, Santos AM, Anzilotti C, Cornall RJ, Davis SJ (2018). Immune checkpoints as therapeutic targets in autoimmunity. Front Immunol.

[14] Funes SC, Manrique de Lara A, Altamirano-Lagos MJ, Mackern-Oberti JP, Escobar-Vera J, Kalergis AM (2019). Immune checkpoints and the regulation of tolerogenicity in dendritic cells: Implications for autoimmunity and immunotherapy. Autoimmun Rev.

[15] Okazaki T, Honjo T (2006). The PD-1-PD-L pathway in immunological tolerance. Trends Immunol.

[16] Topalian SL, Hodi FS, Brahmer JR, Gettinger SN, Smith DC, McDermott DF (2012). Safety, activity, and immune correlates of anti-PD-1 antibody in cancer. N Engl J Med.

[17] Hamid O, Robert C, Daud A, Hodi FS, Hwu WJ, Kefford R (2013). Safety and tumor responses with lambrolizumab (anti-PD-1) in melanoma. N Engl J Med.

[18] Zak KM, Grudnik P, Magiera K, Domling A, Dubin G, Holak TA (2017). Structural biology of the immune checkpoint receptor PD-1 and its ligands PD-L1/PD-L2. Structure.

[19] Panjwani PK, Charu V, DeLisser M, Molina-Kirsch H, Natkunam Y, Zhao S (2018). Programmed death-1 ligands PD-L1 and PD-L2 show distinctive and restricted patterns of expression in lymphoma subtypes. Hum Pathol.

[20] Wu SP, Liao RQ, Tu HY, Wang WJ, Dong ZY, Huang SM (2018). Stromal PD-L1-positive regulatory T cells and PD-1-positive CD8-positive T cells define the response of different subsets of non-small cell lung cancer to PD-1/PD-L1 blockade immunotherapy. J Thorac Oncol.

[21] Sharma P, Allison JP (2015). The future of immune checkpoint therapy. Science.

[22] Yearley JH, Gibson C, Yu N, Moon C, Murphy E, Juco J (2017). PD-L2 expression in human tumors: relevance to anti-PD-1 therapy in cancer. Clin Cancer Res.

[23] Lin X, Lin K, Lin C, Wang J, Tang Y (2020). Prognostic and clinicopathological utility of PD-L2 expression in patients with digestive system cancers: a meta-analysis. Int Immunopharmacol.

[24] Arimatsu Y (1994). Latexin: a molecular marker for regional specification in the neocortex. Neurosci Res.

[25] Liu Q, Yu L, Gao J, Fu Q, Zhang J, Zhang P (2000). Cloning, tissue expression pattern and genomic organization of latexin, a human homologue of rat carboxypeptidase A inhibitor. Mol Biol Rep..

[26] Liang Y, Jansen M, Aronow B, Geiger H, Van Zant G (2007). The quantitative trait gene latexin influences the size of the hematopoietic stem cell population in mice. Nat Genet.

[27] Zhang C, Liang Y (2018). Latexin and hematopoiesis. Curr Opin Hematol.

[28] Kan S, Li R, Tan Y, Yang F, Xu S, Wang L (2022). Latexin deficiency attenuates adipocyte differentiation and protects mice against obesity and metabolic disorders induced by high-fat diet. Cell Death Dis.

[29] Li Y, Huang B, Yang H, Kan S, Yao Y, Liu X (2020). Latexin deficiency in mice up-regulates inflammation and aggravates colitis through HECTD1/Rps3/NF-kappaB pathway. Sci Rep..

[30] Zhang M, Osisami M, Dai J, Keller JM, Escara-Wilke J, Mizokami A (2017). Bone microenvironment changes in latexin expression promote chemoresistance. Mol Cancer Res.

[31] Parra-Torres NM, Cazares-Raga FE, Kouri JB (2014). Proteomic analysis of rat cartilage: the identification of differentially expressed proteins in the early stages of osteoarthritis. Proteome Sci.

[32] Liu Y, Zhang C, Li Z, Wang C, Jia J, Gao T (2017). Latexin inactivation enhances survival and long-term engraftment of hematopoietic stem cells and expands the entire hematopoietic system in mice. Stem cell Rep..

[33] Garcia-Diaz A, Shin DS, Moreno BH, Saco J, Escuin-Ordinas H, Rodriguez GA (2017). Interferon receptor signaling pathways regulating PD-L1 and PD-L2 expression. Cell Rep..

[34] Bromberg J, Wang TC (2009). Inflammation and cancer: IL-6 and STAT3 complete the link. Cancer Cell.

[35] Gamero AM, Young HA, Wiltrout RH (2004). Inactivation of Stat3 in tumor cells: releasing a brake on immune responses against cancer?. Cancer Cell.

[36] Liang Y, Van Zant G (2008). Aging stem cells, latexin, and longevity. Exp Cell Res.

[37] Heinrich F, Lehmbecker A, Raddatz BB, Kegler K, Tipold A, Stein VM (2017). Morphologic, phenotypic, and transcriptomic characterization of classically and alternatively activated canine blood-derived macrophages in vitro. PLoS ONE.

[38] Seed RI, Taurozzi AJ, Wilcock DJ, Nappo G, Erb HHH, Read ML (2019). The putative tumour suppressor protein Latexin is secreted by prostate luminal cells and is downregulated in malignancy. Sci Rep..

[39] Nakamura R, Sene A, Santeford A, Gdoura A, Kubota S, Zapata N (2015). IL10-driven STAT3 signalling in senescent macrophages promotes pathological eye angiogenesis. Nat Commun.

[40] Li J, Diao B, Guo S, Huang X, Yang C, Feng Z (2017). VSIG4 inhibits proinflammatory macrophage activation by reprogramming mitochondrial pyruvate metabolism. Nat Commun.

[41] Ackermann JA, Hofheinz K, Zaiss MM, Kronke G (2017). The double-edged role of 12/15-lipoxygenase during inflammation and immunity. Biochimica Biophysica Acta Mol cell Biol Lipids.

[42] Shirakawa K, Endo J, Kataoka M, Katsumata Y, Yoshida N, Yamamoto T (2018). IL (Interleukin)-10-STAT3-galectin-3 axis is essential for osteopontin-producing reparative macrophage polarization after myocardial infarction. Circulation.

[43] Vasamsetti SB, Karnewar S, Kanugula AK, Thatipalli AR, Kumar JM, Kotamraju S (2015). Metformin inhibits monocyte-to-macrophage differentiation via AMPK-mediated inhibition of STAT3 activation: potential role in atherosclerosis. Diabetes.

[44] Ma W, Zhang J, Guo L, Wang Y, Lu S, Wang Z (2019). Suppressed androgen receptor expression promotes M2 macrophage reprogramming through the STAT3/SOCS3 pathway. EXCLI J.

[45] Akiyama Y, Iizuka A, Kume A, Komiyama M, Urakami K, Ashizawa T (2015). Effect of STAT3 inhibition on the metabolic switch in a highly STAT3-activated lymphoma cell line. Cancer Genomics Proteom.

[46] Messal N, Serriari NE, Pastor S, Nunes JA, Olive D (2011). PD-L2 is expressed on activated human T cells and regulates their function. Mol Immunol.

[47] Wang H, Yao H, Li C, Liang L, Zhang Y, Shi H (2017). PD-L2 expression in colorectal cancer: Independent prognostic effect and targetability by deglycosylation. Oncoimmunology.

[48] Liang CL, Jiang H, Feng W, Liu H, Han L, Chen Y (2021). Total glucosides of paeony ameliorate pristane-induced lupus nephritis by inducing PD-1 ligands(+) macrophages via activating IL-4/STAT6/PD-L2 signaling. Front Immunol.

[49] Tavukcuoglu E, Horzum U, Yilmaz KB, Esendagli G (2020). PD-L2(+) wound zone macrophage-like cells display M1/M2-mixed activation and restrain the effector Th1 responses. Immunol Cell Biol.

[50] Huber S, Hoffmann R, Muskens F, Voehringer D (2010). Alternatively activated macrophages inhibit T-cell proliferation by Stat6-dependent expression of PD-L2. Blood.

