# Supplementary material for: Loss of LXN promotes macrophage M2 polarization and PD-L2 expression contributing cancer immune-escape in mice
Source: Cell Death Discov. 2022 Nov 3;8:440. doi: 10.1038/s41420-022-01227-7 (PMC9630456; doi:10.1038/s41420-022-01227-7)
Supplement: Supplementary file 1 — Supplementary material [file 41420_2022_1227_MOESM1_ESM.docx]

**Supplementary material**

**Loss of LXN Promotes Macrophage M2 Polarization and PD-L2 Expression Contributing Cancer Immune-escape in Mice**

Li Y. *et al*

**Materials and methods**

**Cells and reagents.** All cells were purchased from ATCC, and cultured in Dulbecco’s modified Eagle’s medium (DMEM) in a humidified atmosphere of 5% CO_2_ at 37°C. Dextran sulfate sodium (DSS: CAS 9011-18-1) was purchased from Yeasen Biotech Co., Ltd (HongKong, China). Azoxymethane (AOM: CAS 25843-45-28) was purchased from Sigma-Aldrich (Shanghai, China). Carboxyfluorescein diacetate succinimidyl ester (CFSE, CAS:150347-59-4) was purchased from US Everbright® Inc (Suzhou, China). Antibodies used in this study were shown in Supplementary Table S1. Primers for qPCR were shown in Supplementary Table S2.

**Mouse Model.** *LXN*^-/-^ mice were maintained as F10 generations from *LXN*^+/-^ mice (B6N.129S2-Lxn<tm1Yari>/YariRbrc), which were generated and purchased from RIKEN BioResource Research Center^1,2^. CD45.1 mice are a gift from Zuping Zhou laboratory in Guangxi Normal University. Mouse CRC model was constructed as described previously^3^. Briefly, 10-weeks old WT and *LXN^-/-^* male mice (n=10) were randomly selected and injected intraperitoneally with AOM (10 mg/kg) on day 1. After one week of drinking water, 1.6 % DSS was added to the drinking water for one week, followed by two weeks of DSS-free water. Four cycles of DSS treatment were used. Disease severity or disease activity index (DIA) of colitis-associated disease mouse model was determined based on weight loss, blood in the stool, and stool consistency. The intensity of colitis was monitored and the clinical parameters were evaluated as fellow: anal erosion (score 0-3: 0, normal; 1, mild; 2, moderate; 3, severe), anal bleeding (score 0-3: 0, normal; 1, mild; 2, moderate; 3, severe), diarrhea (score 0-3:0, normal; 1, mild; 2, moderate; 3, severe). Histology was scored in a blinded manner by pathologists. The degree of inflammation was scored as follows: on a scale of 0-3 (0, negative; 1, mild; 2, moderate; 3, severe); damage in crypt architecture was scored as follows: on a scale of 0-4 (0, negative; 1, 0-30% damage to epithelium; 2, 31-65% damage to epithelium; 3, structurally defective epithelium; 4, loss of crypt and epithelium destruction). Tumor grade was assessed by a pathologist blinded to the mouse genotype and treatment, using clinical and pathological scores as described previously^4,5^. The total histopathological score was determined by the summation of the scores from each category. The animal study was performed at the animal facilities of Guangxi Normal University and approved by the Institutional Animal Care and Use Committees. All institutional and national guidelines for the care and use of laboratory animals were followed.

**Isolation of cells from mice.** (1) For cells from tumor tissues, tumor were cut into 2-3mm pieces，the pieces were incubated in digestion medium（digestion medium consistent of RPMI 1640, 5%FBS,1mg/mL collagenase D, 1mg/mL Dnase,1mg/mL hyaluronidase）for 45min at 37℃，and through pass 100µm cell strainers and then centrifuged at 1500rpm for 3min. The remaining pellets were resuspended in 1ml PBS and subjected to Flow-cytometric analysis. (2) For colonic immune cells, colons tissues were isolated, and all the fat, blood vessels were removed. Tumor and colonic tissues were carefully separated and cut into 2-3mm pieces. Small pieces were incubated in digestion medium （digestion medium consistent of RPMI 1640, 5%FBS, 1mg/mL collagenase D, 1mg/mL Dnase, 3mg/mL hyaluronidase）for 2h at 37℃. The suspensions were through pass 100µm cell strainers and then centrifuged at 1500rpm for 3min. The pellet was disrupted and cells were re-suspended in 20mL of 30% Percoll solution above which a 5 mL 80% Percoll solution was positioned, and subjected to gradient centrifugation at 1000 ×g for 30min at 4 °C. The middle layer of cells was collected for further analysis. (3) For bone marrow derived macrophages (BMDM), femurs and tibias were harvested from 6-week-old mice and the marrow was flushed and placed into a sterile suspension of PBS. The bone marrow suspension was cultured in RPMI-1640 medium with 10% FBS and L-glutamine with 50 ng/mL murine M-CSF. The culture medium was changed on day 3 of culture and adherent cells were collected on day 6.

**Subcutaneous tumor models and adoptive transfer of macrophages.** MC38 or LLC cells (5×10^5^ cells/0.1mL PBS) were injected subcutaneously in the flank of 6 to 8-week *WT* and *LXN*^-/-^ mice (n=8). After 7 days, 5×10^6^ CFSE-labelled *WT* or *LXN*^-/-^ macrophages (F4/80^+^CD11b^+^) were intravenous injected at time indicated. Growth of the implanted tumors in *WT* and *LXN*^-/-^ tumor-bearing mice was monitored, and tumor sizes were measured every 3 days. Tumor-bearing mice were anesthetized with isoflurane and photographed by using the IVIS® Spectrum in vivo imaging system.

**Co-cultures of macrophages with T lymphocytes.** The spleen T lymphocytes were isolated and purified by using the murine lymphocyte separation kit (Solarbio) and activated by anti-CD3 (5μg/mL) antibodies for 48 h. The activity of T cells was evaluated by measuring the expression of *IFN-γ*, *IL-2*, and *CD44* by qRT-PCR. T cells (1-5×10^4^ per well) were co-cultured in 96-well plates with a 2:1 ratio of the *WT* or *LXN*^-/-^ BMDMs for 72h. T cells were analyzed by an Attune™ acoustic focusing flow cytometer (ThermoFisher SCIENTIFIC, Shanghai, China).

**Bone marrow transplantation and antibody therapy.** Bone marrow was collected from sex-matched *WT* and *LXN*^-/-^ donor mice (CD45.2) femur and tibia. Recipient mice (CD45.1) were exposed to lethal irradiation with two 5.5 Gy doses (total 11 Gy) at a 4h interval in order to minimize radiation toxicity and then transplanted with 10^7^ bone marrow cells by tail vein injection. After 4 weeks recovery, bone marrow reconstitution was confirmed by analyzing of CD45.2^+^ and CD45.1^+^cells in blood. The transplanted mice were induced with four cycles of AOM/DSS treatment for 13 weeks (n=6). For antibody therapy, MC38 cells (5×105 cells/0.1mL PBS) were engrafted into the subcutaneously of *LXN^-/-^* mice (n=6). After 2 days, these mice were injected intraperitoneally with a 20 mg/kg dose of monoclonal anti-PD-L1, anti-PD-L2, or anti-PD-L1/PD-L2 blocking antibody, IgG (InVivoMab anti-mouse PD-L2 (B7-DC), BE0112-25MG; InVivoMab anti-mouse PD-L1 (B7-H1), BE0101-25MG; InVivoMab mouse IgG2b isotype control, BE0086-25MG) as control antibodies, every 2 days, for a period of 10 days, then tumor tissues were collected and determined.

**Flow cytometric analysis.** For multicolor flow cytometry immunotypic analysis, cells were stained with indicated monoclonal antibodies (Supplementary Table S1) and analyzed on an Attune™ acoustic focusing flow cytometer using Attune software (ThermoFisher SCIENTIFIC, Shanghai, China).

**Plasmid constructs.** *LXN* was amplified by PCR using the primers containing BamHⅠ and EcoRⅠ sites (see Supplementary Table S2). *LXN* was sub-cloned into pFlag-CMV vector.

**Western Blot.** Cells were lysed using RIPA buffer (25 mM Tris-HCl, 150 mM NaCl, 1% Nonidet P-40, 1% sodium deoxycholate, 0.1% SDS, pH 7.6, and proteinase inhibitor mixture). Protein samples were resolved by SDS-PAGE and transferred to nitrocellulose (BioRad).

**Immunohistochemistry staining.** The colon was fixed using 4% paraformaldehyde and embedded in paraffin. For immunohistochemistry staining, tissue was quenched with three hydrogen peroxide, and antigen retrieval was performed in citrate solutions and blocked with PBS solution containing 2% bovine serum albumin (BSA) and 5% goat serum for 1 h at room temperature, followed by the staining with primary antibodies and the corresponding secondary antibodies. Imaging was performed using an Invitrogen™ EVOS™ FL Auto2 microscopy (Thermo Fisher Scientific).

**qRT-PCR.** Total RNAs were extracted from cells or tissues by using TRIzol Reagent (Life Technologies, Rockville, MD) according to the manufacturer’s instructions. qRT-PCR was performed on cDNA from 200 ng of total RNA by using cDNA Synthesis kit and SYBR® Green Master Mix Kit (Exqion). Samples were run on CFX96^TM^ Real-Time system (Bio-Rad). Primer sequences are described in Supplementary Table S2.

**EMSA.** STAT3-DNA binding activity was analyzed by an electrophoretic mobility shift assay (EMSA) using a 5′-biotinylated STAT3 oligonucleotide (5′-GATCCTTCTGGGAATTCCTAGATC-3’; 5’-GATCTAGGAATTCCCAGAAGGATC-3’) (Catalog: GS083A, Beyotime, Shanghai, China). Nuclear extracts were prepared from *WT* and *LXN^-/-^* BMDMs and incubated with the 5′-biotinylated STAT3 oligonucleotide probes according to the recommendations of the EMSA/Gel-Shift Kit (Catalog: GS002, Beyotime, Shanghai, China).

**ChIP.** Formaldehyde-cross-linked chromatin was prepared from *WT* and *LXN^-/-^* BMDMs, and ChIP was performed using the SimpleChIP® Plus Enzymatic Chromatin IP Kit (Magnetic Beads #9005) from CST according to the manufacturer’s instructions. To calculate DNA enrichment in the ChIP assays, PCR was performed in a CFX96^TM^ Real-Time system (Bio-Rad) using the iQ SYBR Green supermix (Bio-Rad) and the primers for the *PD-L1* (F:5’-TTAAGAAGCTATACAATTTCA-3; R:5’-TAGGTAGCATTAGTGAAGATACA-3’) and *PD-L2* promoters (F: 5’-GTGGGTCAGTCACGGTGACAC-3’; R:5’-CTCCTGATCTTGCTTCCACT-3’) were used.

**RNA-seq.** Total RNAs isolated from WT or LXN KO BMDMs using TRIzol reagent (Life Technologies, Rockville, MD). RNA integrity was assessed using the RNA Nano 6000 Assay Kit of the Bioanalyzer 2100 system (Agilent Technologies, CA, USA). The purified RNA samples, with RIN (RNA Integrity Number) over 8.0, determined by Agilent 2100 Bioanalyzer (Agilent, Waldbroon, Germany) were sequenced at Novogene corporation (Beijing, China). The clustering of the index-coded samples was performed on a cBot Cluster Generation System using TruSeq PE Cluster Kit v3-cBot-HS (Illumia) according to the manufacturer’s instructions. After cluster generation, the library preparations were sequenced on an Illumina Novaseq platform and 150 bp paired-end reads were generated. Differential expression analysis of two conditions/groups (two biological replicates per condition) was performed using the DESeq2 R package (1.16.1). Genes with an adjusted *P*-value <0.05 found by DESeq2 were assigned as differentially expressed.

**Statistical analyses.** Data analysis was performed using Prism 8 (GraphPad Software). Data are expressed as means ± SEM and the n values for each data set are provided in the figure legends. The statistical significance of differences was assessed by 2-tailed Student’s t-test except where otherwise indicated.

**References**

1. Arimatsu Y. Latexin: a molecular marker for regional specification in the neocortex. Neuroscience research 1994, 20(2): 131-135.
2. Li Y, Huang B, Yang H, Kan S, Yao Y, Liu X, et al. Latexin deficiency in mice up-regulates inflammation and aggravates colitis through HECTD1/Rps3/NF-kappaB pathway. Scientific reports 2020, 10(1): 9868.
3. Wang T, Fan C, Yao A, Xu X, Zheng G, You Y, et al. The Adaptor Protein CARD9 Protects against Colon Cancer by Restricting Mycobiota-Mediated Expansion of Myeloid-Derived Suppressor Cells. Immunity 2018, 49(3): 504-514 e504.
4. Wirtz S, Popp V, Kindermann M, Gerlach K, Weigmann B, Fichtner-Feigl S, et al. Chemically induced mouse models of acute and chronic intestinal inflammation. Nature protocols 2017, 12(7): 1295-1309.
5. 34. Kargl J, Haybaeck J, Stancic A, Andersen L, Marsche G, Heinemann A, et al. O-1602, an atypical cannabinoid, inhibits tumor growth in colitis-associated colon cancer through multiple mechanisms. Journal of molecular medicine 2013, 91(4): 449-458.
