# Supplementary material for: Loss of LXN promotes macrophage M2 polarization and PD-L2 expression contributing cancer immune-escape in mice
Source: Cell Death Discov. 2022 Nov 3;8:440. doi: 10.1038/s41420-022-01227-7 (PMC9630456; doi:10.1038/s41420-022-01227-7)
Supplement: Supplementary file 2 — Supplementary Figures [file 41420_2022_1227_MOESM2_ESM.docx]

**Supplementary Figure and Figure Legends**


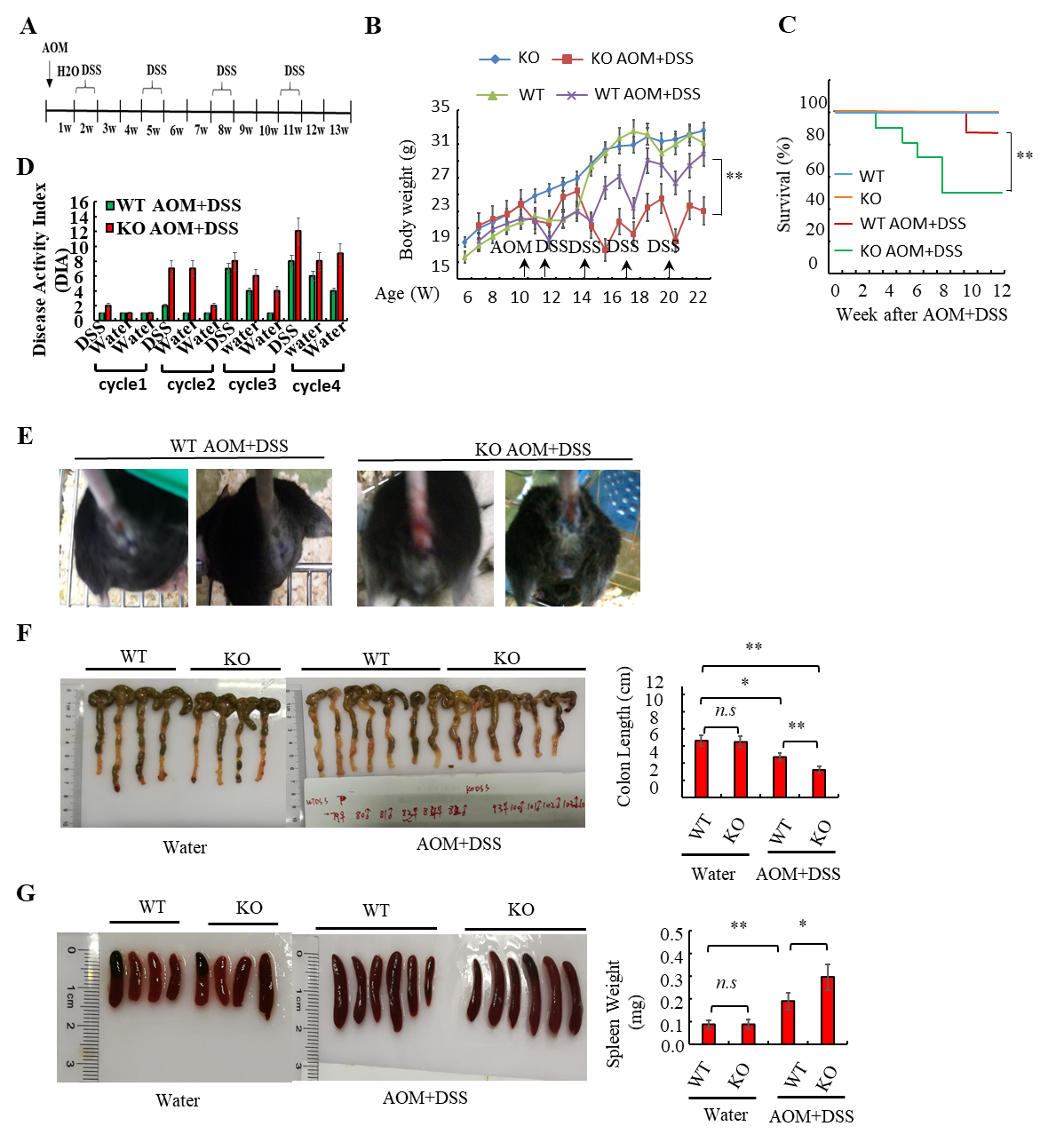


**Figure S1. Evaluation of physiological indexes of WT and KO mice treated with AOM/DSS.** (**A**) Representative the experimental protocol for the induction of colitis-associated cancer in LXN^-/-^ (KO) and littermate LXN^+/+^ (WT) mice. (**B-D)** Representative the body weight (B), mice survival (C), and disease activity index (DIA) (**D**) of mice during AOM/DSS treatment. n=10, ***P*<0.01. (**E)** Representative the anal bleeding of WT and KO mice after treated with AOM/DSS. (**F, G**) Representative the colon length (F) and spleen weight (G) from WT and KO mice treated or untreated with AOM/DSS. n=6, **P*<0.05, ***P*<0.01, n.s, no significance.
