# Supplementary material for: Loss of LXN promotes macrophage M2 polarization and PD-L2 expression contributing cancer immune-escape in mice
Source: Cell Death Discov. 2022 Nov 3;8:440. doi: 10.1038/s41420-022-01227-7 (PMC9630456; doi:10.1038/s41420-022-01227-7)
Supplement: Supplementary file 3 — Supplementary Table S1 [file 41420_2022_1227_MOESM3_ESM.docx]

**Table S1. Information of antibodies**

| **Name** | **Supplier** | **Catalog** | **WB** | **IF** | **IHC** | **IP** | **Flow cytometric** |
| --- | --- | --- | --- | --- | --- | --- | --- |
| **LXN** | **Sino Biological** | **10211-R101** | **1:1000** | **1:200** | **1:100** | **1:100** |  |
| **GAPDH** | **ZSGB-BIO** | **TA-08** | **1:1000** |  |  |  |  |
| **β-actin** | **ZSGB-BIO** | **TA-09** | **1:1000** |  |  |  |  |
| **IgG** | **Beyotime** | **A7016** |  |  |  | **1:100** |  |
| **Flag** | **Sino Biological** | **101274-MM05** | **1:1000** |  |  | **1:40** |  |
| **Myc** | **Sino Biological** | **100029-MM08** | **1:5000** |  |  | **1:100** |  |
| **JAK1** | **CST** | **3332S** | **1:1000** |  |  |  |  |
| **p-JAK1** | **Beyotime** | **AF5857** | **1:1000** |  |  |  |  |
| **STAT3** | **eBioscience** | **9D8** | **1:5000** |  |  |  |  |
| **pY705-STAT3** | **Beyotime** | **PA5-85445** | **1:2000** | **1:200-** |  |  |  |
| **pS727-STAT3** | **Beyotime** | **44-384G** | **1:2000** |  |  |  |  |
| **B220, PE- Cyanine5.5** | **eBioscienc** | **35-0452-82** |  |  |  |  | **0.125 µg/test** |
| **CD3, APC** | **eBioscienc** | **17-0032-82** |  |  |  |  | **0.5 µg/test** |
| **CD3, Alexa Fluor 700** | **eBioscienc** | **56-0032-82** |  |  |  |  | **0.25 µg/test** |
| **CD8, PE-Cyanine7** | **eBioscienc** | **25-0081-82** |  |  |  |  | **0.5 µg/test** |
| **CD4, PE** | **eBioscienc** | **12-0041-82** |  |  |  |  | **0.125 µg/test** |
| **CD11b, PE-Cyanine7** | **eBioscienc** | **25-0112-82** |  |  |  |  | **0.125 µg/test** |
| **F4/80, eFluor 450** | **eBioscienc** | **48-4801-82** |  |  |  |  | **0.5 µg/test** |
| **CD16/32, PerCP-Cyanine5.5** | **eBioscienc** | **45-0161-82** |  |  |  |  | **0.125 µg/test** |
| **CD206, APC** | **eBioscienc** | **17-2061-82** |  |  |  |  | **0.25 µg/test** |
| **CD163, Super Bright 702** | **eBioscienc** | **67-1631-82** |  |  |  |  | **0.25 µg/test** |
| **PD-L2, PE** | **eBioscienc** | **12-5986-82** |  |  |  |  | **0.5 µg/test** |
| **PD-L2** | **eBioscienc** | **PA5-20344** | **0.5µg/mL** | **20µg/mL** |  |  |  |
| **PD-L1** | **Beyotime** | **AF7710** | **1:1000** |  |  |  |  |
| **CD163** | **Beyotim** | **AF6453** | **1:1000** |  |  |  |  |
| **CD3e** | **eBioscienc** | **16-0031-38** |  |  |  |  |  |
| **CD11c, PE-eFluor 610** | **eBioscienc** | **61-0114-82** |  |  |  |  | **0.25 µg/test** |
| **CD45, eFluor 506** | **eBioscienc** | **69-0451-82** |  |  |  |  | **0.5 µg/test** |
| **CD45.1, FITC** | **eBioscienc** | **11-0453-82** |  |  |  |  | **0.5 µg/test** |
| **CD45.2, eFluor 506** | **eBioscienc** | **69-0454-82** |  |  |  |  | **0.25 µg/test** |
| **Sca1 - PE-Cy7** | **eBioscienc** | **25-5981-82** |  |  |  |  | **0.25 µg/test** |
| **ckit-APC-eFluor™ 780** | **eBioscienc** | **47-1171-82** |  |  |  |  | **0.25 µg/test** |
