# Supplementary material for: Loss of LXN promotes macrophage M2 polarization and PD-L2 expression contributing cancer immune-escape in mice
Source: Cell Death Discov. 2022 Nov 3;8:440. doi: 10.1038/s41420-022-01227-7 (PMC9630456; doi:10.1038/s41420-022-01227-7)
Supplement: Supplementary file 4 — Supplementary Table S2 [file 41420_2022_1227_MOESM4_ESM.docx]

**Table S2. Primer list**

| **For qPCR** | |
| --- | --- |
| **Primer** | **Sequence (5’-3’)** |
| LXN | Forward, 5’-GCGGTTATGTAATGTGGCAG-3’; Reverse, 5’-AATGTCGTGGAGTAGAATGGTG-3’ |
| IL-10 | Forward, 5’-GCTGGACAACATACTGCTAACC-3’; Reverse, 5’-ATTTCCGATAAGGCTTGGCAA-3’ |
| CD163 | Forward, 5’-ATGGGTGGACACAGAATGGTT-3’; Reverse, 5’-CAGGAGCGTTAGTGACAGCAG-3’ |
| IL-6 | Forward,5’-TACCACTTCACAAGTCGGAGGC-3’; Reverse,5’-CTGCAAGTGCATCATCGTTGTTC-3’ |
| IL-1β | Forward,5’-TGCCACCTTTTGACAGTGATG-3’; Reverse,5’-TGATGTGCTGCTGCGAGATT-3’ |
| GAPDH | Forward,5’-AGGTCGGTGTGAACGGATTTG-3’; Reverse,5’-TGTAGACCATGTAGTTGAGGTCA-3’ |
| PD-L2 | Forward,5’-CTGCCGATACTGAACCTGAGC-3’; Reverse,5’-GCGGTCAAAATCGCACTCC-3’ |
| PD-L1 | Forward, 5’-GCTCCAAAGGACTTGTACGTG-3’; Reverse, 5’-TGATCTGAAGGGCAGCATTTC-3’ |
| PD-1 | Forward, 5’-ACCCTGGTCATTCACTTGGG-3’; Reverse, 5’-CATTTGCTCCCTCTGACACTG-3’ |
| CD44 | Forward, 5’-TCGATTTGAATGTAACCTGCCG-3’; Reverse, 5’-CAGTCCGGGAGATACTGTAGC-3’ |
| INF-ɤ | Forward, 5’-TGCGGGGTTGTATCTGGG-3’; Reverse, 5’-CTGGCCCGGAGTGTAGACAT-3’ |
| IL-2 | Forward, 5’-TGAGCAGGATGGAGAATTACAGG-3’; Reverse, 5’-GTCCAAGTTCATCTTCTAGGCAC-3’ |
| Ccl2 | Forward, 5’- TTAAAAACCTGGATCGGAACCAA -3’; Reverse, 5’- GCATTAGCTTCAGATTTACGGGT -3’ |
| iNOS | Forward, 5’-GTTCTCAGCCCAACAATACAAGA-3’; Reverse, 5’-GTGGACGGGTCGATGTCAC-3’ |
| Arg1 | Forward, 5’-CTCCAAGCCAAAGTCCTTAGAG-3’; Reverse, 5’-AGGAGCTGTCATTAGGGACATC-3’ |
| **For plasmid construction** | |
| LXN | Forward,5’-CACGAATTCAATGGAAATCCCGCCGA-3’; Reverse,5’- TCTAGAATCCTTCCAGTTGTACTTCC-3’ |
