# Supplementary figures and images for: Loss of LXN promotes macrophage M2 polarization and PD-L2 expression contributing cancer immune-escape in mice
Source: Cell Death Discov. 2022 Nov 3;8:440. doi: 10.1038/s41420-022-01227-7 (PMC9630456; doi:10.1038/s41420-022-01227-7)

Uncropped western bolt gels used in the main figures


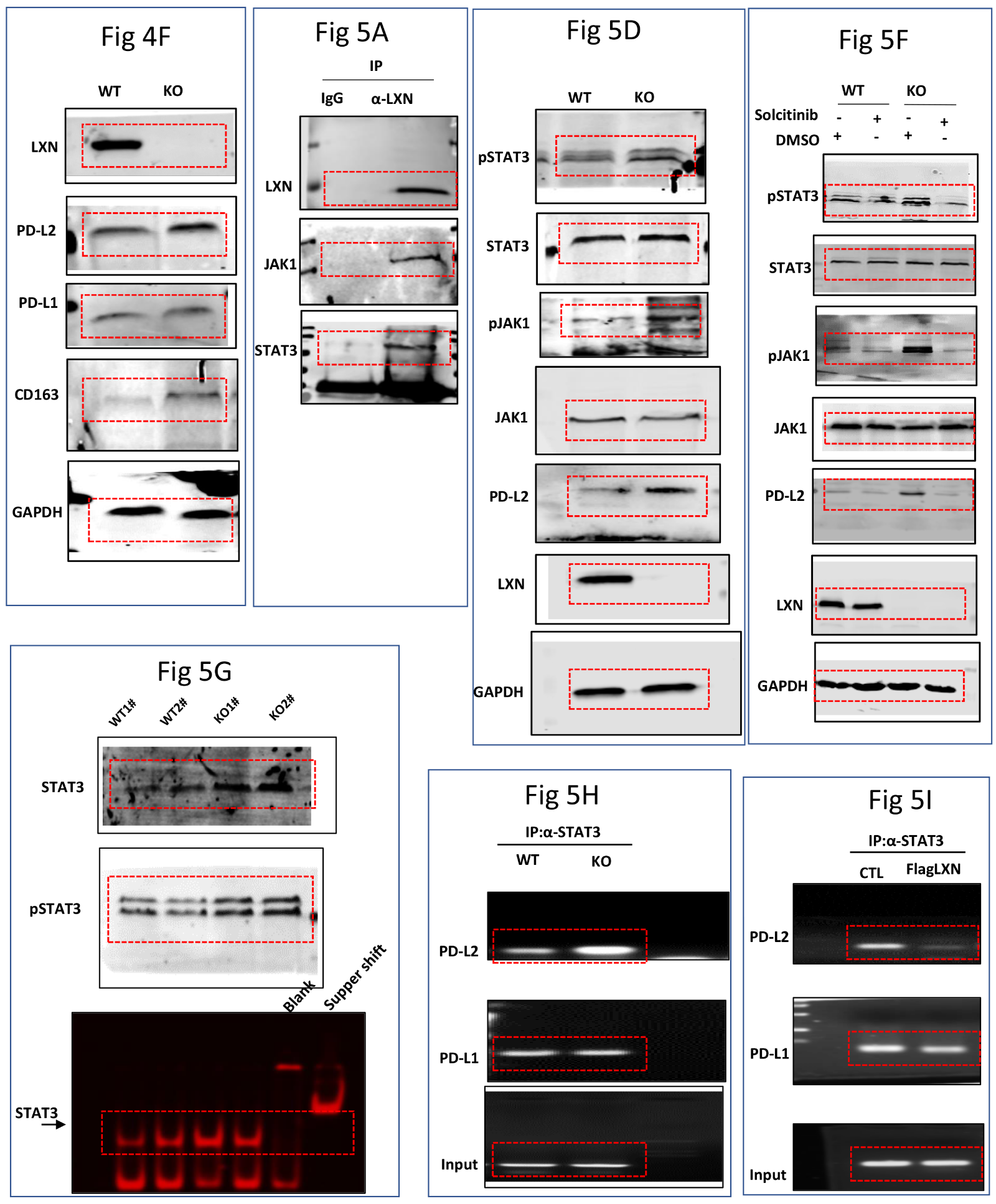

Supplement: Supplementary file 6 — Original Data File [file 41420_2022_1227_MOESM6_ESM.docx]
